# Supplementary material for: EBP50 Depletion and Nuclear β-Catenin Accumulation Engender Aggressive Behavior of Colorectal Carcinoma through Induction of Tumor Budding
Source: Cancers (Basel). 2023 Dec 29;16(1):183. doi: 10.3390/cancers16010183 (PMC10778391; doi:10.3390/cancers16010183)
Supplement: Supplementary file 1 [file cancers-16-00183-s001.zip › Supplementary Figures S1-S4.docx]

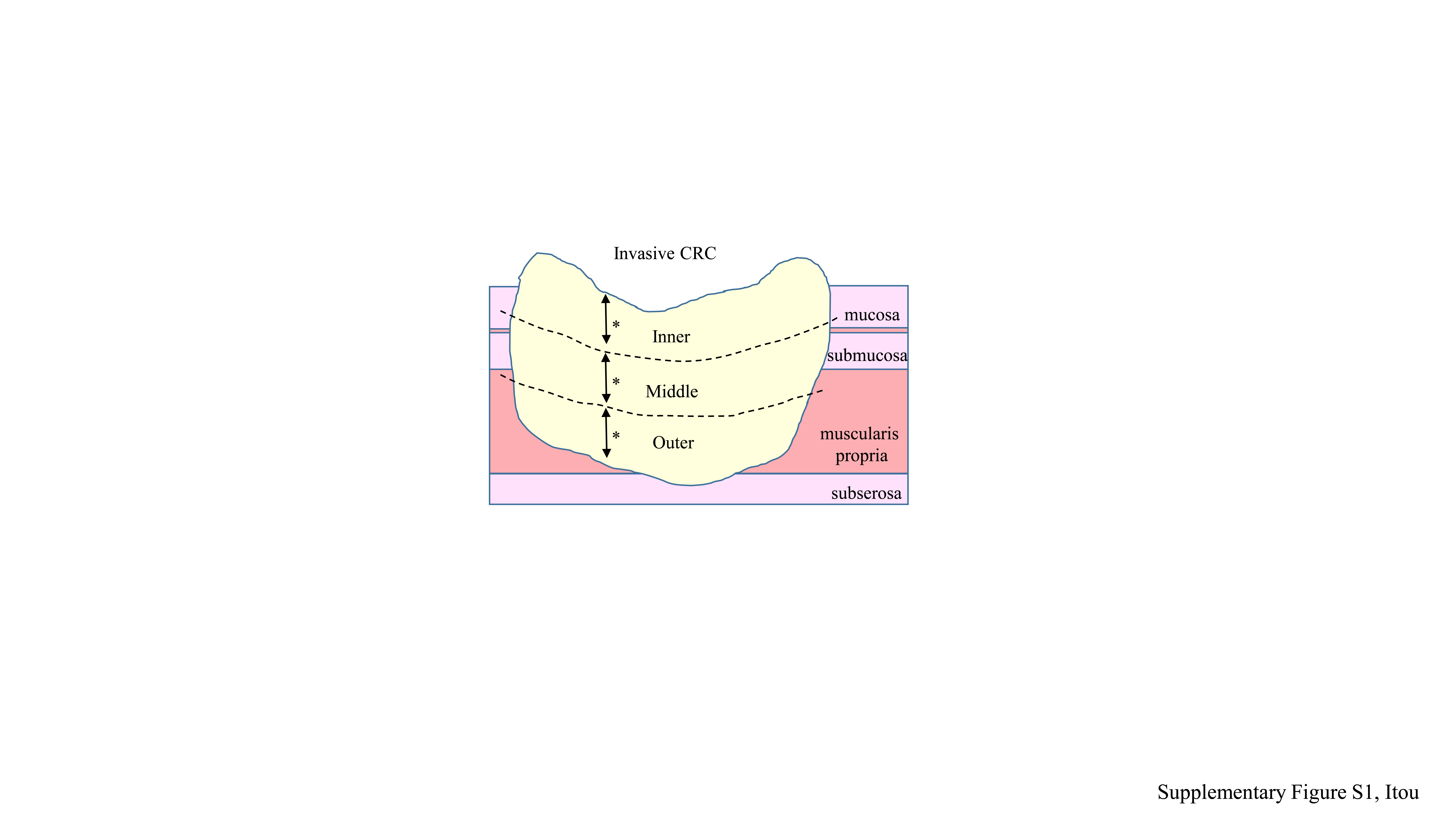


**Supplementary Figure S1.** A diagram showing the three subclassifications of inner, middle, and outer parts of a tumor lesion on the basis of tumor size. *, one-third of the lesions.


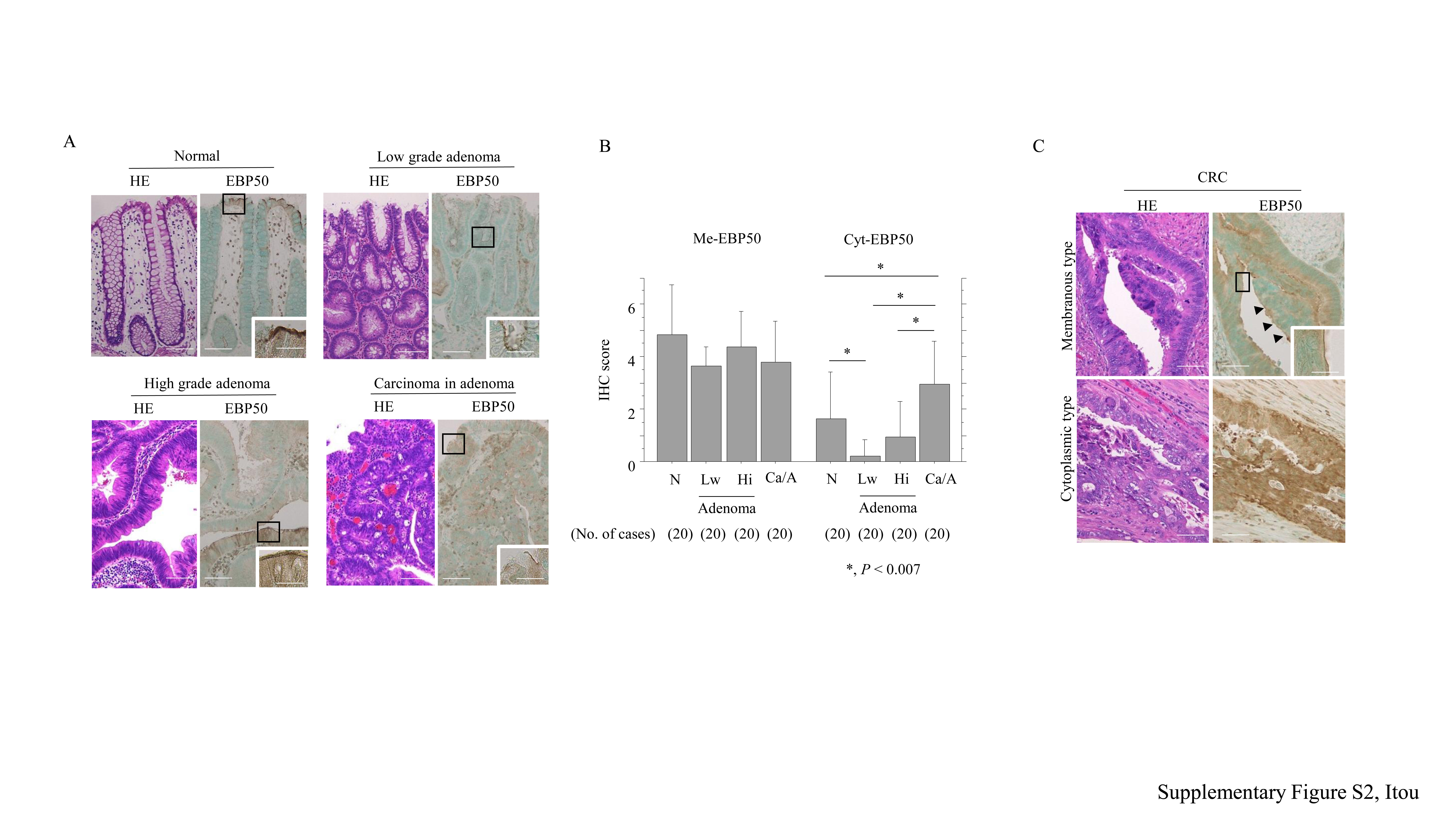


**Supplementary Figure S2. EBP50 expression during adenoma-carcinoma progression in CRC.** (A) Staining with HE and IHC for EBP50 in normal colorectal mucosa (left upper), low- and high-grade adenomas (right upper and left lower), and carcinoma in adenoma (right lower). The closed boxes are magnified in insets. Original magnification, x100 and x400 (inset). Scale bar = 50 μm and 20 μm (insets). (B) IHC scores for the indicated proteins in normal (N), low (Lw) and high (Hi) grade adenomas, and carcinoma in adenoma (Ca/A) lesions. The scores shown are means ± SDs. Statistical analyses were carried out using the Mann-Whitney *U*-test. Me, membranous, Cyt, cytoplasmic. (C) Staining with HE and IHC for EBP in membranous (upper) and cytoplasmic (lower) EBP50 expression in CRC. Note the EBP50 immunoreactivity at the apical plasma membrane (indicated by arrowheads in the upper right panel). The closed boxes are magnified in insets. Original magnification, x100 and x400 (inset). Scale bar = 30 μm and 15 μm (inset).


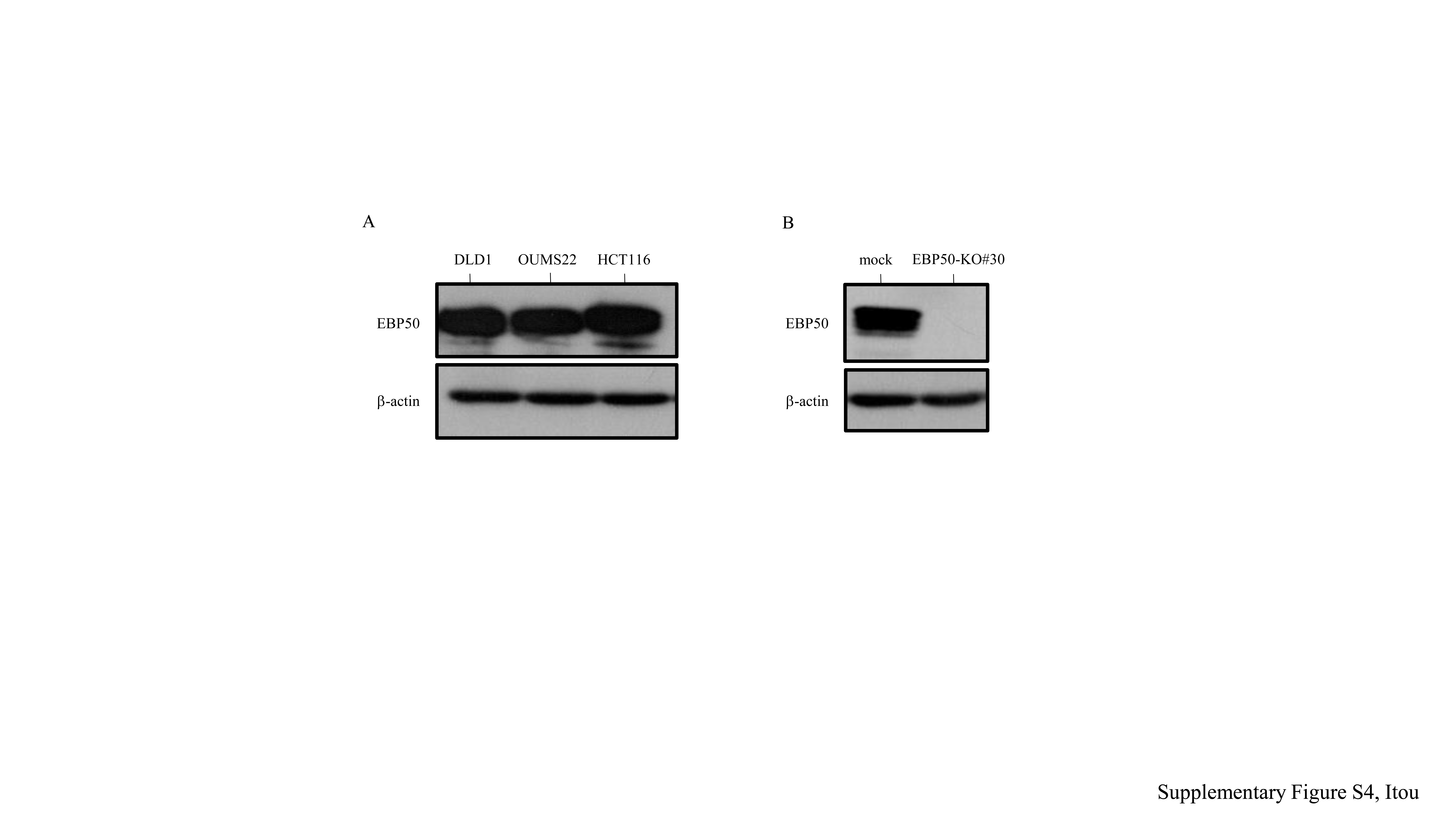


**Supplementary Figure S3.** (A,B) Western blot analysis for the indicated proteins in total lysates from the indicated cell lines.


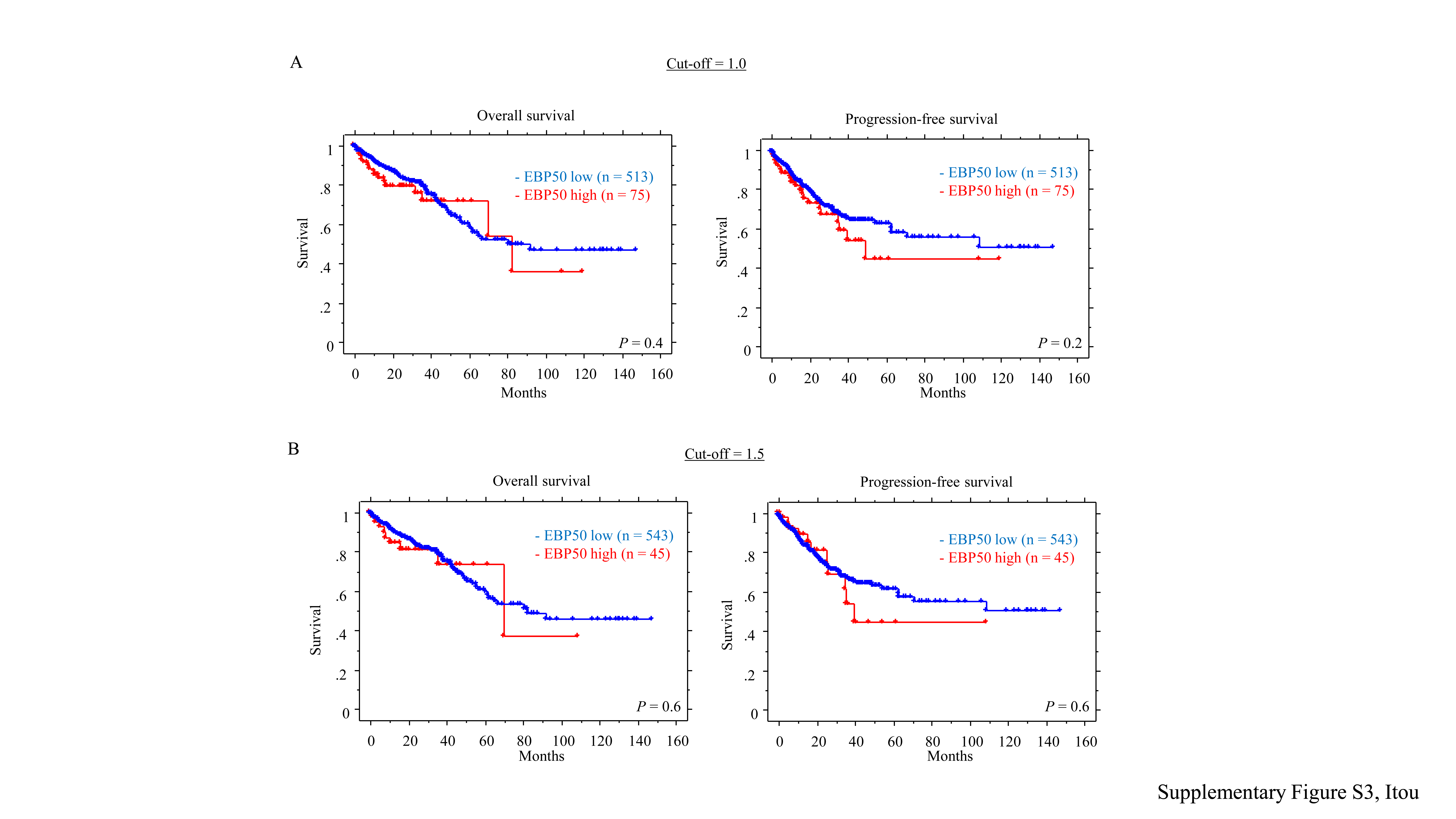


**Supplementary Figure S4.** TCGA data analysis for associations between EBP50 status and prognosis in CRC. OS (left) and PFS (right) relative to EBP50 mRNA using cut-off values, 1.0 (A) and 1.5 (B). n, number of cases. Statistical analyses were performed using the log rank test.
