# Supplementary figures and images for: EBP50 Depletion and Nuclear β-Catenin Accumulation Engender Aggressive Behavior of Colorectal Carcinoma through Induction of Tumor Budding
Source: Cancers (Basel). 2023 Dec 29;16(1):183. doi: 10.3390/cancers16010183 (PMC10778391; doi:10.3390/cancers16010183)

Figure 3C

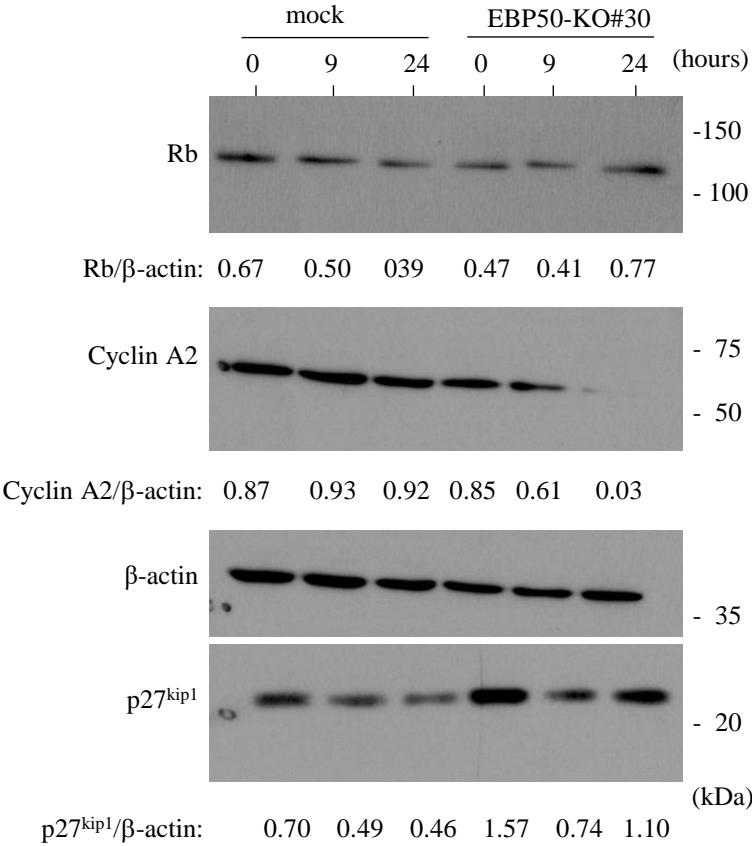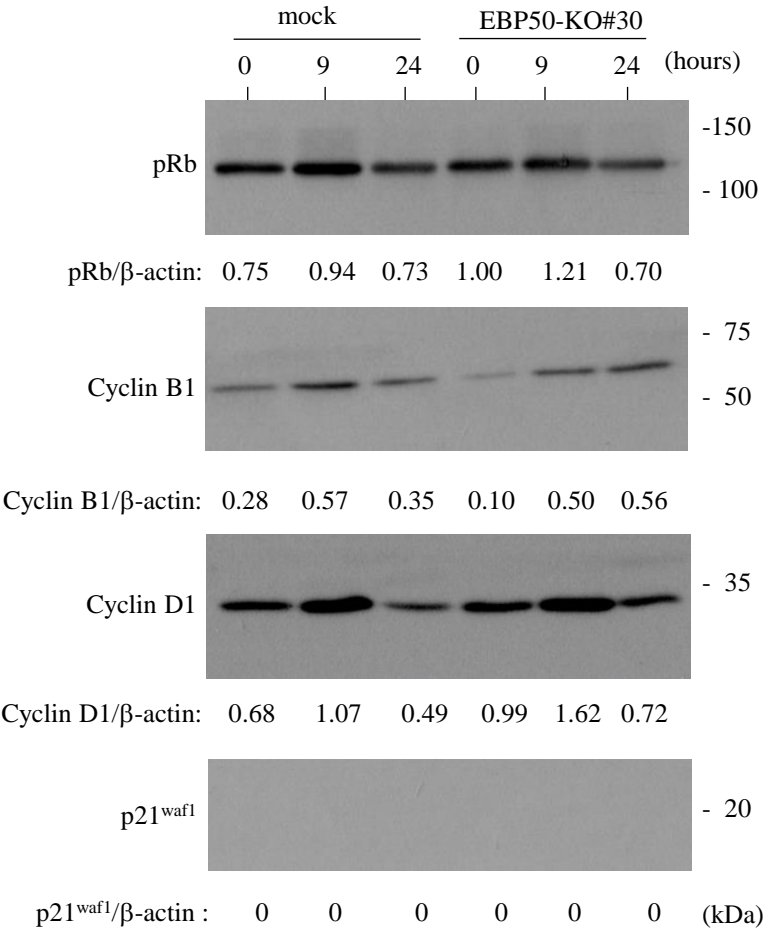

Figure 4C

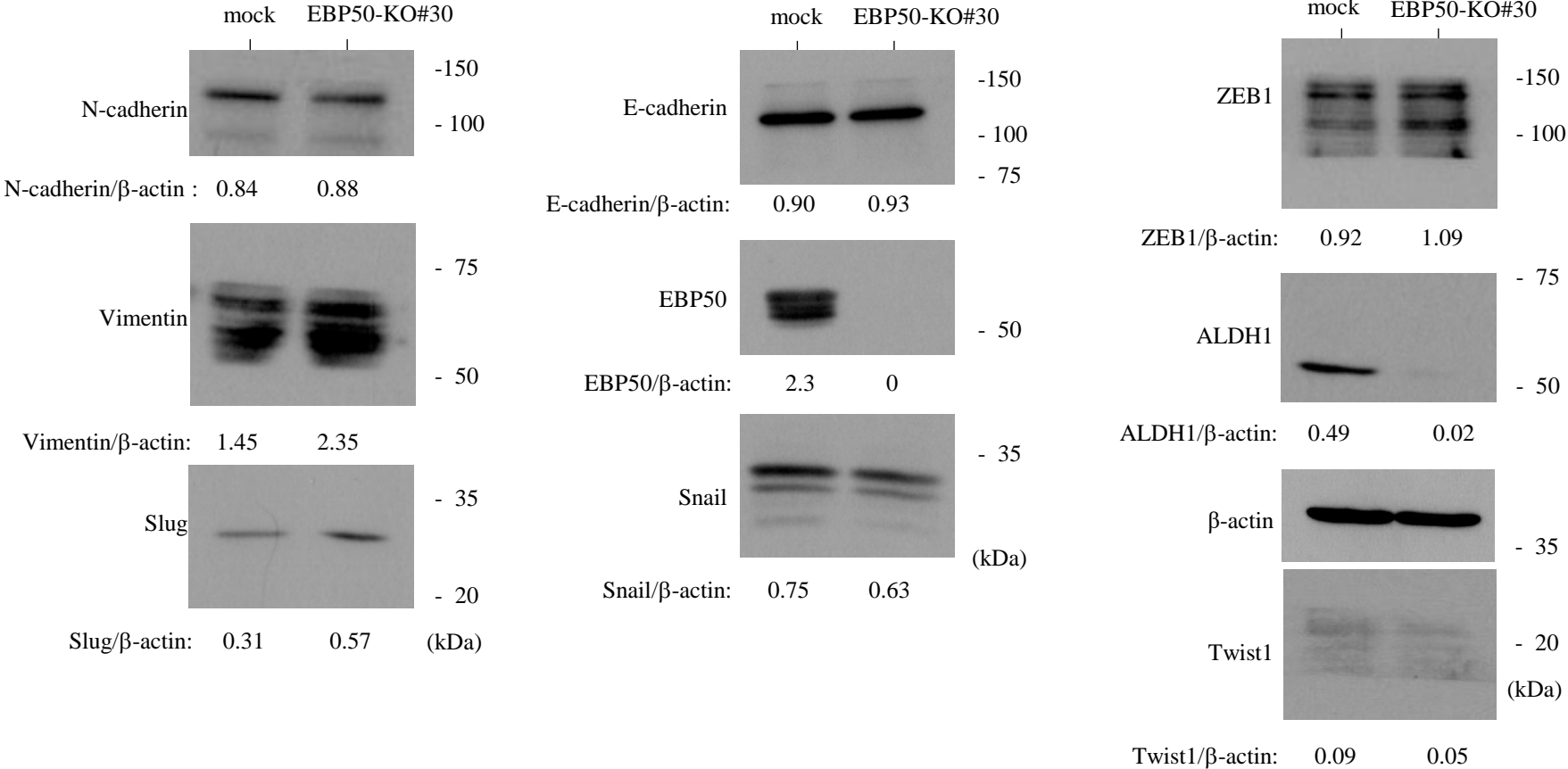

Figure 5A

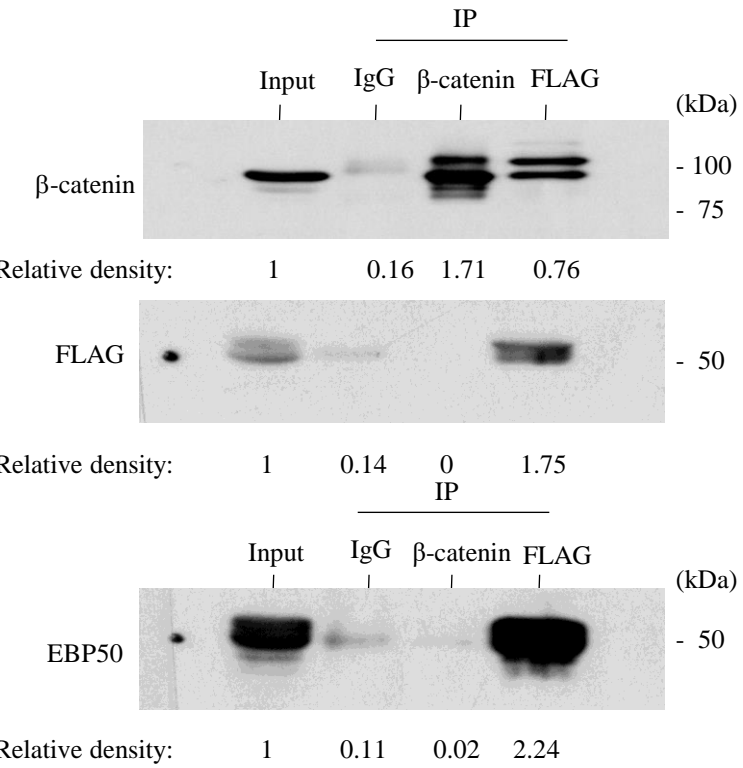

Figure 5C

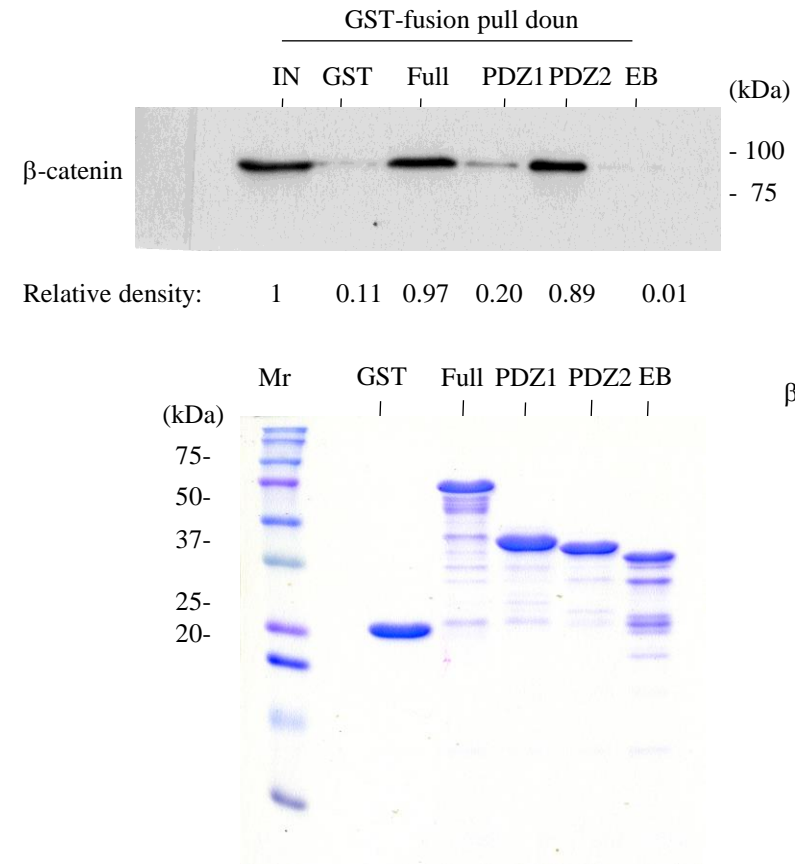

Figure 5F

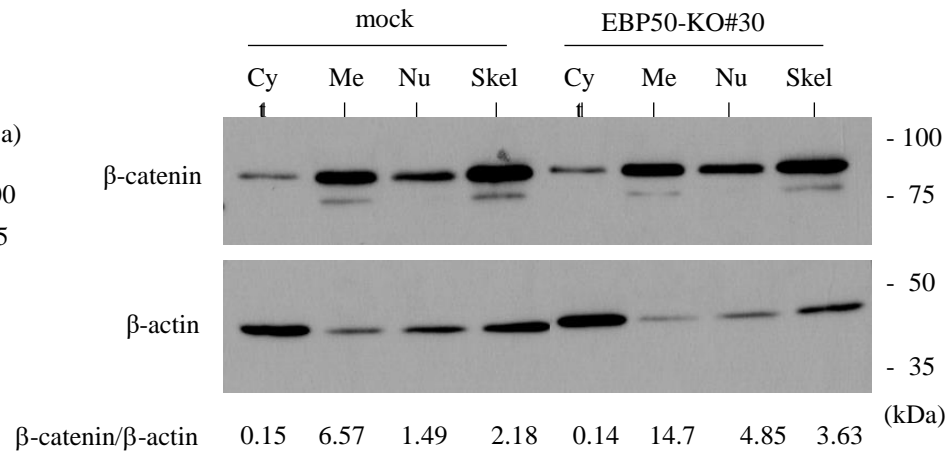

Supplementary Figure S3

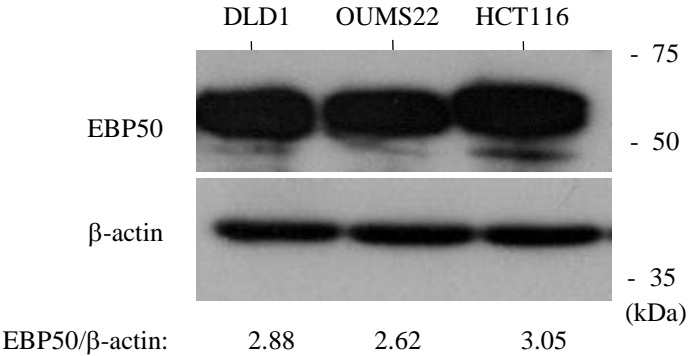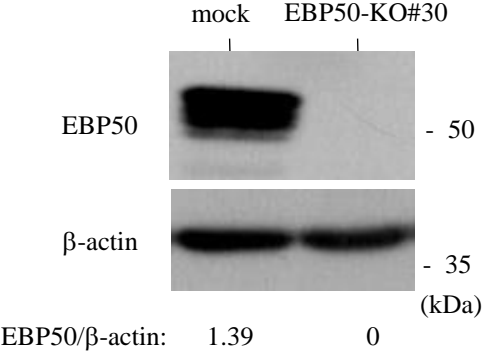

Supplement: Supplementary file 1 [file cancers-16-00183-s001.zip › File S1--EBP Colon WB raw data.pdf]
